# Social support and sleep quality in people with schizophrenia living in the community: the mediating roles of anxiety and depression symptoms

**DOI:** 10.3389/fpubh.2024.1414868

**Published:** 2024-07-30

**Authors:** Xin Liu, Chao Li, Xushu Chen, Fengxiang Tian, Juan Liu, Yuanyuan Liu, Xiang Liu, Xiaolan Yin, Xiangrui Wu, Chuanlong Zuo, Changjiu He

**Affiliations:** ^1^School of Nursing, Chengdu Medical College, Chengdu, China; ^2^The Fourth People’s Hospital of Chengdu, Chengdu, China; ^3^Western Theater General Hospital, Chengdu, China; ^4^Department of Epidemiology and Biostatistics, West China School of Public Health and West China Fourth Hospital, Sichuan University, Chengdu, Sichuan, China

**Keywords:** anxiety, depression, sleep quality, social support, schizophrenia

## Abstract

**Introduction:**

Research has demonstrated that higher social support is associated with better psychological health, quality of life, cognition, activities of daily living, and social participation, but the relationship between social support and sleep quality remains unknown. This study aims to investigate the mediating effects of anxiety and depression in the relationship between social support and sleep among community-dwelling patients with schizophrenia.

**Method:**

Purposive sampling was used to collect face-to-face data from 1,107 community-dwelling patients with schizophrenia in Chengdu, Sichuan Province, China, between April and July 2023. The Athens Insomnia Scale (AIS) was used to assess sleep quality; the Generalized Anxiety Disorder 7-item scale (GAD-7) was utilized to evaluate anxiety symptoms; and the Patient Health Questionnaire-9 (PHQ-9) was employed to assess depressive symptoms. The mediating effect of anxiety and depression symptoms was assessed using the bootstrap method via Model 6 (Serial multiple mediator model) of the SPSS PROCESS macro.

**Results:**

Among the 1,107 participants, the proportions of people with schizophrenia experiencing anxiety, depressive symptoms, and poor sleep quality were 22.8, 37.7, and 42.1%, respectively. Mediation analyses indicated that although social support had no direct effect on sleep quality, anxiety and depressive symptoms fully mediated the relationship between social support and sleep quality.

**Conclusion:**

Patients with schizophrenia experience low levels of social support and poor sleep quality. To enhance the sleep quality of individuals with schizophrenia, all levels of society (government, medical institutions, and communities) must pay more attention to mental health. Implementing diverse intervention measures to strengthen social support and improve symptoms of anxiety and depression should be considered. This approach may potentially lead to an improvement in sleep quality among individuals with schizophrenia.

## 1 Introduction

The sleep issue is a global public health issue of great concern. According to studies, the prevalence of sleep disturbance in the general population ranges from 9.2 to 15.5%, negatively affecting people’s ability to function socially and their overall quality of life (1, 2). Sleep disturbance is common in psychiatric disorders (3). Approximately 30–80% of individuals with schizophrenia suffer from some form of disturbed sleep, such as difficulties initiating or maintaining sleep (4). The most obvious manifestations of sleep abnormalities among patients with schizophrenia include increased sleep latency, reduced total sleep time, and reduced sleep efficiency (5).

Research has shown that the association between sleep quality and schizophrenia is bidirectional (6). People with schizophrenia often experience poor sleep quality, and poor sleep quality may exacerbate schizophrenia symptoms (7). Sleep problems in schizophrenia are associated with worse health outcomes (8, 9), including cognitive dysfunction (10), more severe psychiatric disorders (11), metabolic disturbances (12), and impaired quality of life (13). Evidence suggests that targeted insomnia interventions can help reduce psychiatric symptoms and improve functional outcomes (14). These findings highlight the importance of screening and managing sleep problems in people with schizophrenia. Moreover, it is essential to investigate the potential modifiable factors that influence or facilitate sleep.

The reasons behind the poor sleep quality experienced by patients with schizophrenia are diverse and can be complex. Research has identified multiple factors that impact the sleep quality of individuals with schizophrenia. Known risk factors encompass sociodemographic variables (15), disease-related factors (16), lifestyle choices (9), environmental influences (17), and psychosocial aspects (18). In recent years, psychosocial factors have garnered extensive attention as potential modifiable elements. Psychological factors can mitigate sleep disturbances through the self-regulation of emotions, thereby reducing the prevalence of sleep issues. However, current research primarily focuses on negative psychological indicators, such as anxiety and depression, which are common comorbidities in schizophrenia (19, 20). Not only do they contribute to the worsening of schizophrenia’s primary symptoms, but they also have a detrimental impact on sleep quality (21). The interrelation between poor sleep, heightened anxiety, and increased depression creates a vicious cycle, further impairing individuals’ ability to engage with their treatment and recovery (22, 23). There is a limited understanding of how protective psychosocial factors, such as social support, influence sleep quality in patients with schizophrenia.

Social support is a multidimensional concept, typically referring to the receipt of material or emotional support and assistance from others (24). Social support can directly protect mental health through the benefits of social relationships, or it can serve as a buffer against external pressures, thereby indirectly safeguarding psychological well-being (25). Research indicates that high levels of social support can enhance individuals’ ability to cope with stress and alleviate anxiety and depression by improving sleep quality (26, 27).

Research suggests that the relationship between perceived social support and sleep quality is influenced by the impact of social support on reducing adverse psychological reactions, such as anxiety and depression, and improving health status (28). Specifically, poor sleep quality is associated with higher levels of anxiety and depression, whereas higher levels of social support are linked to reductions in these symptoms, thereby enhancing sleep quality (29, 30). Similarly, research among medical personnel highlighted the importance of social support in buffering against work-related stressors, ultimately leading to improved mental health and sleep outcomes (31). Additionally, studies of medical students found that anxiety and depression mediate the relationship between perceived stress and sleep quality (32). In stroke patients, anxiety and depression also play a mediating role between social support and sleep quality (33). While the relationship between social support and sleep quality has been explored in various populations, less attention has been given to community-dwelling individuals with schizophrenia. Moreover, the potential mediating roles of anxiety and depression symptoms in this relationship remain underexplored. Understanding these mediating mechanisms is crucial, as it can inform targeted interventions to improve sleep quality and overall well-being in this vulnerable population.

This study addresses a critical gap in the literature by elucidating the complex interactions between social support, sleep quality, and psychological symptoms in schizophrenia. By identifying the mediating roles of anxiety and depression, our research offers potential pathways for interventions aimed at enhancing social support mechanisms to improve sleep quality and reduce psychological distress among community-dwelling schizophrenics. Thus, this study contributes to broader efforts to improve mental health outcomes and quality of life for individuals living with schizophrenia.

In this article, we propose a comprehensive model to illustrate how social support is associated with sleep quality. The main hypotheses to be tested in this integrated model are: (1) social support was negatively related to sleep quality; (2) social support was negatively related to anxiety symptoms; (3) social support was negatively related to depression symptoms; (4) anxiety symptoms were positively related to sleep quality; and (5) depression symptoms were positively related to sleep quality. This study is based on a cross-sectional survey of the mental health of patients with schizophrenia in communities in Chengdu, China, with the aim of testing a comprehensive model of social support, anxiety, depression, and sleep quality.

## 2 Methods

### 2.1 Participants and study design

This was a cross-sectional study conducted from April to July 2023. Prior to the commencement of this study, we had obtained approval from the Ethics Committee of West China Fourth Hospital, Sichuan University. The study employed a convenience sampling method to recruit participants from communities in Chengdu, Sichuan Province, China. Convenience sampling is a specific type of non-probability sampling method that relies on collecting data from populations that are conveniently available to participate in the study, and it is most commonly used in quantitative research (34). It is most commonly used in quantitative studies. A total of 1,107 patients with schizophrenia were recruited, with the inclusion criteria being: (1) meets the diagnostic criteria for schizophrenia according to the Tenth Revision of the International Statistical Classification of Diseases and Related Health Problems (ICD-10); and (2) consents to participate in this study. The exclusion criteria were as follows: (1) presence of other severe mental disorders, organic brain diseases, or mental retardation; (2) refusal to sign the informed consent form; and (3) communication disorders.

### 2.2 Measurements

#### 2.2.1 Basic sociodemographic data

Participants provided demographic information about their name, age, gender, education level, marital status, living situation, annual household income, and living alone status.

#### 2.2.2 Social support rating scale

The 10-item Social Support Rating Scale (SSRS) was used to assess the level of social support in patients with schizophrenia (35). The scale consists of three dimensions: subjective support, objective support and support utilization. Subjective support: This dimension assesses perceived emotional and informational support from family and friends (Items 2, 6, 7). Objective support: This dimension includes items that measure tangible support such as financial aid and material assistance (Items 1, 3, 4, 5). Support utilization: This dimension evaluates how well participants utilize the available support (Items 8 to 10). Each entry is categorized into 4 levels in ascending order, with corresponding scores of 1, 2, 3, and 4. The total scores ranged from 12 to 66, with higher scores being associated with higher levels of social support, and the specific scores corresponding to the levels are shown in Table 1. The SSRS has been widely applied to the Chinese population and has been proven to possess good internal consistency. Cronbach’s alpha coefficient was determined to be 0.820 (36).

**Table 1:** Social support scale rating evaluation.

Total score range	Evaluation level
12–22	Low
23–44	Medium
45–66	High

#### 2.2.3 Anxiety symptoms

The Generalized Anxiety Disorder 7-item (GAD-7) scale is employed for screening and assessing symptoms of anxiety (37). Comprising seven items, it evaluates the frequency of seven symptoms experienced by patients over the past two weeks, such as “difficulty relaxing” or “excessive worry.” The response categories range from “not at all,” “several days,” “more than half the days,” to “nearly every day,” with corresponding scores of 0, 1, 2, and 3. Total scores ranged from 0 to 21, with higher scores being associated with higher levels of anxiety, and scores of 5 or higher being considered indicative of anxiety symptoms, with specific scores corresponding to levels shown in Table 2. Due to a Cronbach’s alpha value of 0.879, the GAD-7 demonstrates a high level of internal consistency in the current sample (38).

**Table 2:** Generalized anxiety disorder 7-item evaluation.

Total score range	Evaluation level
0–4	No symptoms
5–9	Mild anxiety
10–14	Moderate anxiety
15–21	Severe anxiety

#### 2.2.4 Depressive symptoms

The Patient Health Questionnaire-9 (PHQ-9) is utilized for the screening and assessment of depressive symptoms (39, 40). It comprises nine items that evaluate the frequency of nine symptoms experienced by patients over the past 2 weeks, including depressed mood and anhedonia. The response categories are “not at all,” “several days,” “more than half the days,” and “nearly every day,” scored as 0, 1, 2, and 3, respectively. Total scores ranged from 0 to 27, with higher scores being associated with greater levels of depression, and scores of 5 or higher being considered indicative of depressive symptoms, with specific scores corresponding to levels shown in Table 3. With a Cronbach’s alpha value of 0.89, the PHQ-9 demonstrates a high level of internal consistency in the current sample. The reliability and validity of the PHQ-9 in the general population, as well as in patients with mental disorders, have been demonstrated (40, 41).

**Table 3:** Patient health questionnaire-9 evaluation.

Total score range	Evaluation level
0–4	No symptoms
5–9	Mild depression
10–14	Moderate depression
15–19	Moderately severe depression
20–27	Severe depression

#### 2.2.5 Insomnia

The Athens Insomnia Scale (AIS) is used to record the severity of insomnia among all participants (42). The AIS consists of 8 items, including 5 items related to difficulties falling asleep and 3 items related to daytime dysfunction, to assess sleep quality over the past month. The response categories are “no problem,” “minor problem,” “considerable problem,” and “severe problem,” scored as 0, 1, 2, and 3, respectively. The total score ranged from 0 to 24, with higher scores being associated with poorer sleep quality, and the specific scores corresponding to the levels are shown in Table 4. The AIS in the current sample has internal consistency (Cronbach’s alpha, 0.805).

**Table 4:** Athens insomnia scale evaluation.

Total score range	Evaluation level
0–3	Normal
4–6	Suspected insomnia
7–24	Insomnia

### 2.3 Statistical analysis

The data were entered using EpiData software, with two individuals independently entering and consistently coding the questionnaires to ensure data quality. All analyses were conducted using IBM SPSS Statistics version 26.0 (Armonk, NY, USA). A two-tailed significance level was set at *α* = 0.05. Continuous variables were presented as mean ± standard deviation, while categorical variables were expressed as frequencies (n) and percentages (%). Pearson’s correlation analysis was used to test the correlation between social support, anxiety, depression, and sleep, where the variables input are the scores obtained according to the corresponding scales, which are continuous variables. We used the four-step mediation effects test proposed by (43) to analyze mediation effects based on hierarchical multiple regression. The method provides clear steps, is easy to implement, and can significantly improve the identification and interpretation of the role of mediating variables, thus providing a comprehensive understanding of the mediating mechanism. Firstly, the preliminary mediation effect was determined through the four-step test: (1) testing the relationship between the independent variable (social support) and the dependent variable (sleep quality); (2) testing the relationship between the independent variable (social support) and the mediator variables (anxiety and depression); (3) testing the relationship between the mediator variables (anxiety, depression) and the dependent variable (sleep quality); and (4) testing whether the relationship between the independent variable and the dependent variable significantly weakens or becomes non-significant after adding the mediator variables. In the analysis, we applied the statistical method developed by Preacher and Hayes, known as the Bootstrap method for mediating effect testing (specifically Model 6). This method was used to examine whether a mediating variable influences the relationship between an independent variable and a dependent variable. To ensure the robustness and accuracy of their results, we performed 5,000 iterations (“resamples”) of the data. From these iterations, we calculated a 95% confidence interval (CI) that is corrected for any potential biases, providing a range within which the true mediating effect is likely to fall. In addition, we also randomly selected subsets from the data to similarly perform mediated effects analysis to again verify the robustness of the results.

## 3 Results

### 3.1 Common method bias test

In this study, the self-assessment scale was used for data collection, and in order to avoid the influence of the process of stating the questions or the respondents’ social expectations or emotions, the Harman one-factor test was used for the common method bias test (44). When the explained variance rate of the first factor in the Harman one-factor test results exceeds 40%, it can be assumed that there is a large bias in the results of the common method. The explained rate of the first factor in this study is 17.98%, which is lower than 40% and below the test standard, and it can be assumed that there is no serious common method bias effect in the results of this questionnaire.

### 3.2 Demographic characteristics

The general demographic characteristics of these 1,107 community-dwelling patients with schizophrenia are shown in Table 5. The final participant sample of this study included 1,107 community-dwelling schizophrenia patients, with an average age of 50.7 years. There were 536 male (48.4%) and 571 female (51.6%) participants who responded to the survey. The majority of participants had an educational level of junior high school or above (74.4). The marital status of most participants was married (46.4%). The household registration of the majority of participants was urban (69.4%). The family monthly income for the majority of participants ranged from 20,000 to 40,000 yuan (25.3%). The majority of participants lived with others (88.4%).

**Table 5:** Demographic characteristics of the responders (*n* =  1,107).

Variables		*Means* ± SD
Continuous variables		
Age (years)		50.7 ± 12.68
Categorical variables		No (%)
Gender	Male	536 (48.4)
	Female	571 (51.6)
Education level	Primary school or below	283 (25.6)
	Junior school and above	824 (74.4)
Marital status	Single	381 (34.4)
	Married	514 (46.4)
	Divorce	173 (15.6)
	Widowhood	39 (3.5)
Household registration	Urban	768 (69.4)
	Rural	339 (30.6)
Annual household income	<10,000	118 (10.7)
	10,000–20,000	221 (20.0)
	20,000–40,000	280 (25.3)
	40,000–60,000	195 (17.6)
	60,000–80,000	102 (9.2)
	80,000–100,000	57 (5.1)
	>100,000	57 (5.1)
	Prefer to no answer	77 (7.0)
Living state	Live alone	128 (11.6)
	Live with others	979 (88.4)

SD, Stand deviation.

### 3.3 Prevalence of symptoms of anxiety, depression, insomnia, and the level of social support

**Figure 1:**
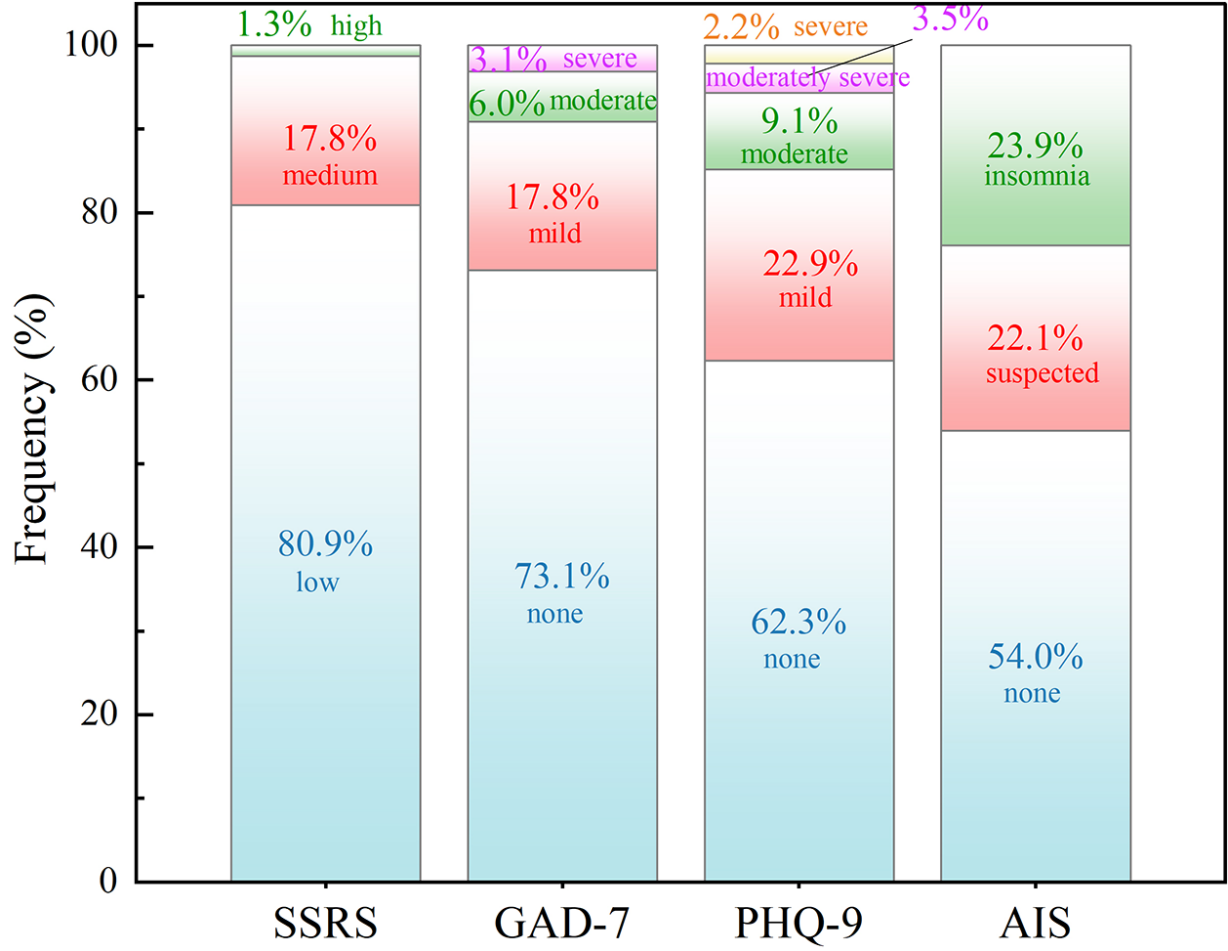
Level of social support and prevalence of anxiety, depression and insomnia among patients with schizophrenia.

Overall, the proportion of participants reporting mild, moderate and severe anxiety symptoms was 17.8, 6.0, and 3.1%, respectively. The proportion of participants reporting mild, moderate, moderately severe, and severe depressive symptoms was 22.9, 9.1, 3.5, and 2.2%, respectively. The proportions of participants reporting suspected insomnia and insomnia were 22.1 and 23.9%. In addition, the participants with low, medium, and high levels of social support accounted for 80.9, 17.8, and 1.3%, respectively (Figure 1).

### 3.4 Correlation between the main study variables

**Figure 2:**
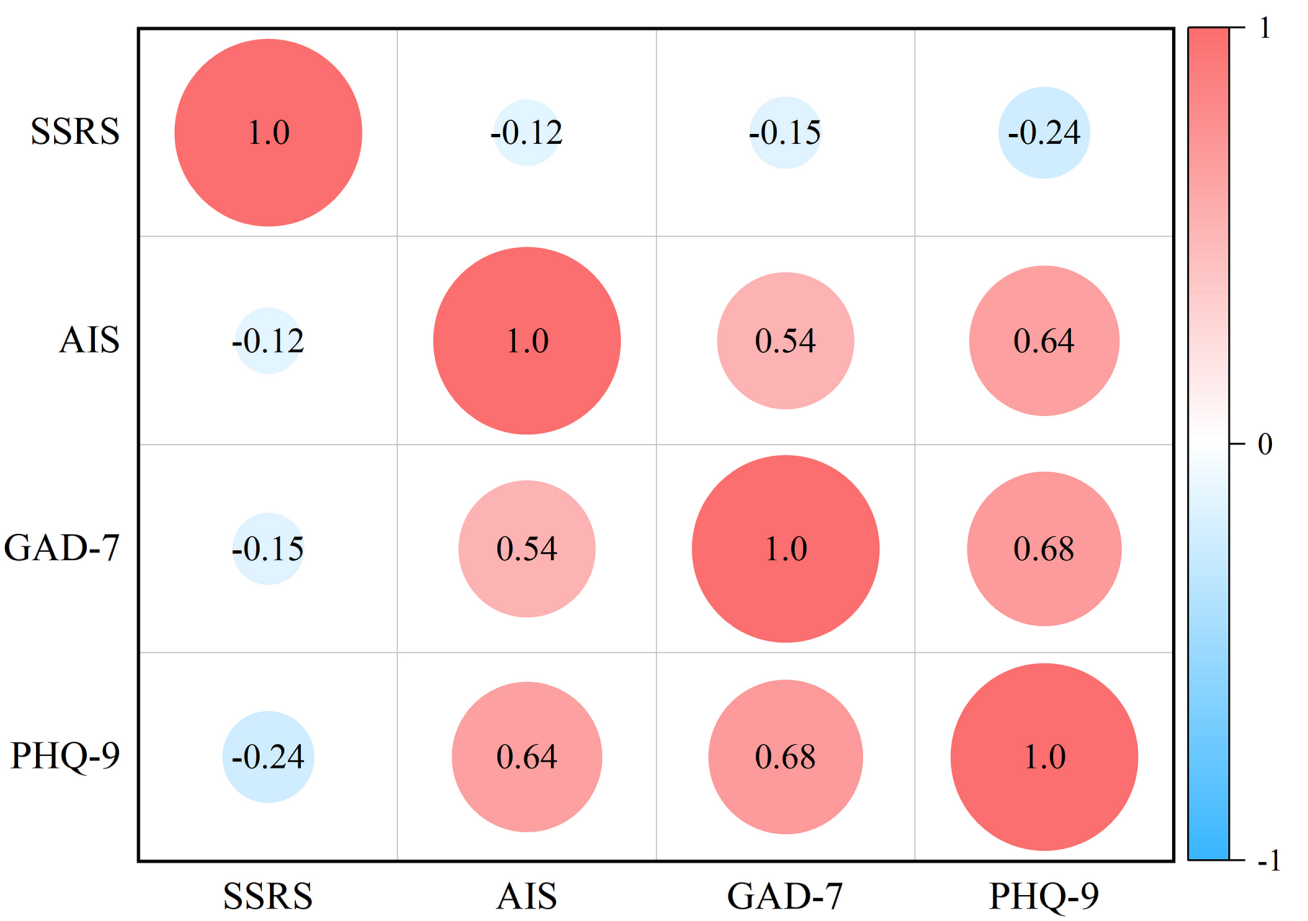
Correlation between the main study variables.

The Pearson product–moment correlation analysis (Figure 2) showed that social support was negatively correlated with sleep quality (*r* = −0.125, *p* < 0.01), anxiety symptoms (*r* = −0.148, *p* < 0.01) and depression symptoms (*r* = −0.239, *p* < 0.01). Moreover, statistically significant positive correlations were observed between sleep quality and anxiety (*r* = 0.536, *p* < 0.01), and depression symptoms (*r* = 0.641, *p* < 0.01).

### 3.5 Mediating effects of anxiety and depression in the relationship between social support and sleep quality

**Figure 3:**
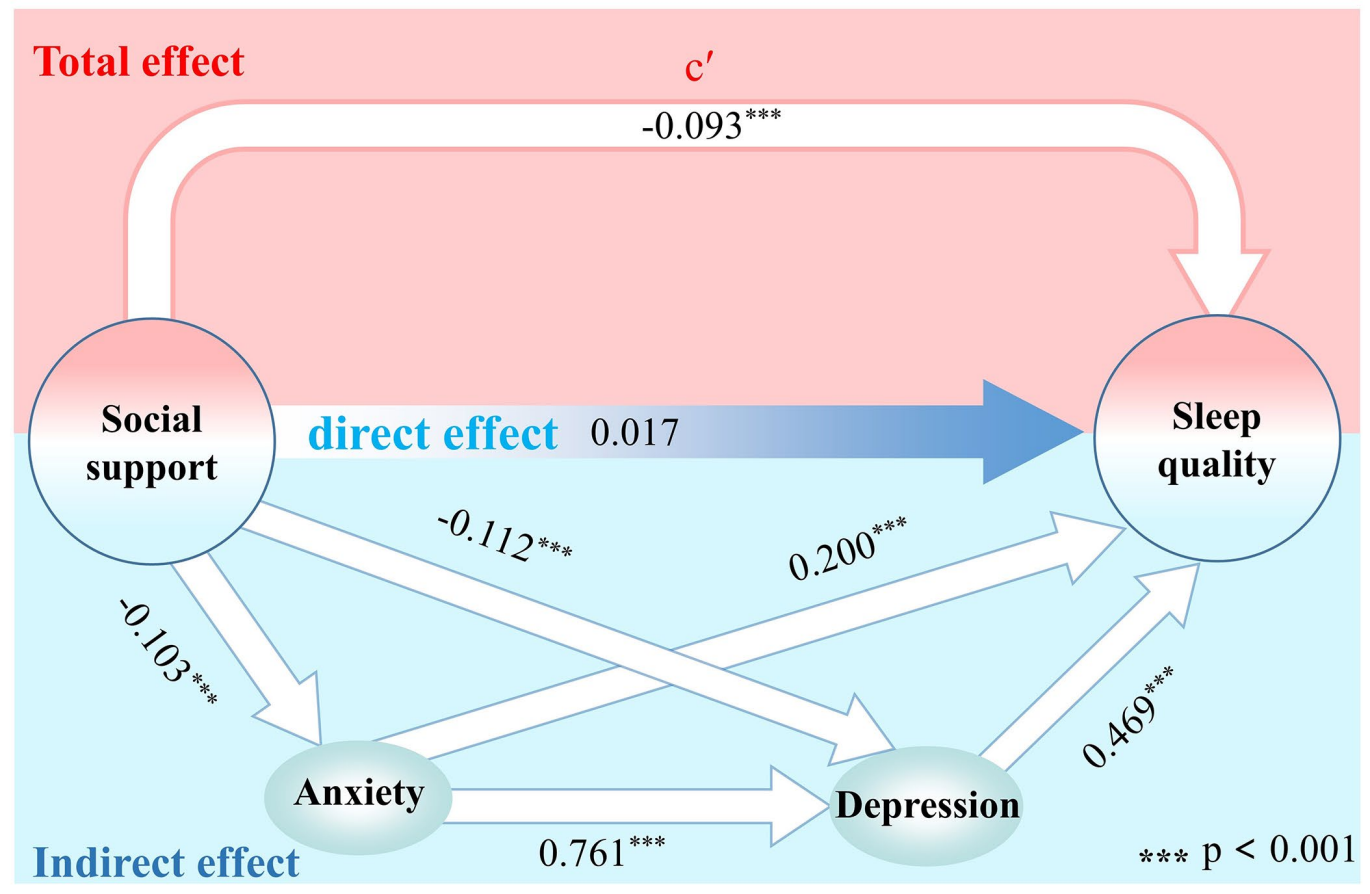
Mediation model of anxiety and depression symptoms between social support and sleep quality.

As shown in Table 6, Model 1 is a linear regression between each control variable and anxiety, which shows that, except for age, there is no linear regression relationship between demographic control variables and anxiety. Model 2 is the addition of the variable of social support to Model 1, which revealed that, social support had a significant negative effect on anxiety (*β* = −0.103, *p* < 0.001). Similarly, Model 3 revealed a linear regression relationship between demographic control variables and sleep quality. After controlling for the effect of demographic variables, Model 4 revealed a significant negative effect of social support with sleep quality (*β* = −0.093, *p* < 0.001). Model 5 further added the anxiety variable to the model, at which point anxiety showed a significant effect on sleep quality (*β* = 0.587, *p* < 0.001), while the effect of social support on sleep quality was no longer significant (*β* = −0.036, *p* > 0.05), i.e., the effect of social support on sleep quality was no longer significant due to the presence of anxiety. These preliminary validated that anxiety plays a fully mediating role between social support and sleep quality. Model 6 added depression as a dependent variable to model 5, and the regression results showed that anxiety had a significant effect on sleep quality (*β* = 0.200, *p* < 0.001), and depression had a significant effect on sleep quality (*β* = 0.469, *p* < 0.001), however, at this time, the effect of social support on sleep quality was not significant (*β* = 0.017, *p* > 0.05), which verified that the role of anxiety and depression in the fully mediating role between social support and sleep quality (Figure 3).

**Table 6:** Mediation of anxiety tested by multivariate hierarchical regression.

Variables	Anxiety		Sleep quality			
	Model 1	Model 2	Model 3	Model 4	Model 5	Model 6
Constant term	3.577	6.001	1.047	3.240	−0.104	−2.492
Control variables						
Gender	0.740	0.873	0.963	1.083	0.597	0.521
Age	−0.007*	−0.009	0.042	0.041	0.045	0.042
Education level	−0.081	−0.045	−0.227	−0.195	−0.170	−0.127
Marital status	−0.039	0.029	0.020	0.042	0.026	0.006
Household registration	−0.189	−0.023	0.348	0.434	0.447	0.561
Annual household income	−0.094	−0.170	0.051	0.068	0.162	0.129
Living state	0.325	−0.385	0.698	0.057	0.271	0.496
Independent variable						
Social support		−0.103***		−0.093***	−0.036	0.017
Intermediate variable						
Anxiety					0.557***	0.200***
Depression						0.469***
*R* ^2^	0.016	0.040	0.037	0.054	0.321	0.457
Δ*R*^2^	0.016	0.024	0.037	0.017	0.267	0.135
F	2.633	5.735***	5.964	7.818***	57.677***	92.090***

*p < 0.05, ***p < 0.001.

As shown in Table 7, Model 1 is a linear regression between each control variable and depression, and other demographic control variables have no linear regression relationship with depression. Model 2 is the addition of the variable of social support to Model 1, and the results showed that, social support had a significant negative effect on depression (*β* = −0.190, *p* < 0.001). Similarly, Model 3 revealed a linear regression relationship between demographic control variables and sleep quality. After controlling for the effect of demographic variables, Model 4 revealed a significant negative effect of social support with sleep quality (*β* = −0.093, *p* < 0.001). Model 5 further added the depression variable to the model, at which point depression showed a significant effect on sleep quality (*β* = 0.587, *p* < 0.001), while the effect of social support on sleep quality was no longer significant (*β* = 0.018, *p* > 0.05), i.e., the effect of social support on sleep quality was no longer significant due to the presence of depression. These preliminary validated that depression plays a fully mediating role between social support and sleep quality. Model 6 adds depression as a dependent variable to model 5, and the regression results show that depression has a significant effect on sleep quality (*β* = 0.469, *p* < 0.001), and anxiety has a significant effect on sleep quality (*β* = 0.200, *p* < 0.001), however, at this time, the effect of social support on sleep quality is not significant (*β* = 0.017, *p* > 0.05), which verifies that the effect of anxiety and depression in the fully mediating role between social support and sleep quality.

**Table 7:** Mediation of depression tested by multivariate hierarchical regression.

Variables	Depression		Sleep quality			
	Model 1	Model 2	Model 3	Model 4	Model 5	Model 6
Constant term	5.187	9.663	1.047	3.240	−2.429	−2.492
Control variables						
Gender	0.580	0.825	0.963	1.083	0.599	0.521
Age	0.003	0.001	0.042	0.041	0.040	0.042
Education level	−0.191	−0.124	−0.227	−0.195	−0.122	−0.127
Marital status	−0.062	0.063	−0.020	0.042	0.004	0.006
Household registration	−0.437	−0.262	0.348	0.434	0.587	0.561
Annual household income	−0.093	−0.059	0.051	0.068	0.102	0.129
Living state	0.535	−0.773	0.698	0.057	0.510	0.496
Independent variable						
Social support		−0.190***		−0.093***	0.018	0.017
Intermediate variable						
Depression					0.587***	0.469***
Anxiety						0.200***
*R* ^2^	0.008	0.068	0.037	0.054	0.438	0.457
Δ*R*^2^	0.008	0.060	0.037	0.017	0.384	0.019
F	1.291	10.073***	5.964	7.818***	94.853***	92.090***

***p < 0.001.

As shown in Table 8, after controlling for demographic variables that may have influenced the results, the mediating effects of anxiety and depression between social support and sleep quality were significant, with 95% CI excluding 0. The mediating effects were specifically generated through 3 paths. Path 1 was social support → anxiety → sleep quality, with a significant mediating effect of anxiety, accounting for 17.8% of the total indirect effect; path 2 was social support → depression → sleep quality, with a significant mediating effect of depression, accounting for 48.5% of the total indirect effect; and path 3 was social support → anxiety → depression → sleep quality, with a significant chained mediating effect of anxiety and depression, accounting for 33.7% of the total indirect effect. The direct effect of social support on sleep quality was not significant, which indicates that anxiety and depression play a fully chain-mediated role in the effect of social support on sleep. In addition, the results of the subset mediated effects analyses are presented in the Supplementary File. Similarly, the results suggest that anxiety and depression play a fully mediated role in the effects of social support on sleep quality.

**Table 8:** Mediation analysis of social support and sleep quality (*N* = 1,107).

Path		Effect	Boot SE	95%CI		Effect ratio
				LL	UL	
Total effect		−0.093	0.021	−0.134	−0.052	
Direct effect		0.017	0.016	−0.015	0.049	
Indirect effect	Total indirect effect	−0.110	0.012	−0.134	−0.086	100%
	Social support→ anxiety → sleep quality	−0.021	0.006	−0.034	−0.086	17.8%
	Social support→ depression → sleep quality	−0.052	0.009	−0.071	−0.037	48.5%
	Social support→ anxiety →depression→ sleep quality	−0.037	−0.007	−0.051	−0.024	33.7%

SE, Standard Error; CI, Confidence interval; LLCI, Lower Limit Confidence interval; ULCI, Upper Limit Confidence interval.

## 4 Discussion

This study aims to explore the relationships between social support, anxiety, depression, and sleep quality among schizophrenia patients living in communities in Chengdu, China. The main focus of our attention is the effect of social support on sleep quality in psychiatric patients and dedicated to explaining the role that anxiety and depression play in this. The results of correlation and hierarchical multiple regression analyses showed that social support has a significant effect on sleep quality among community-dwelling schizophrenia patients directly. However, the effect of social support on sleep quality was no longer significant when both anxiety and depression are included, highlighting the complex interactions between mental health and sleep in this population.

The results of this study, as shown by correlation analysis and multivariate hierarchical analysis, indicated a significant positive correlation between the symptoms of anxiety and depression and the degree of insomnia in schizophrenia patients. Specifically, symptoms of anxiety and depression were directly associated with poor sleep quality in schizophrenia patients, which is consistent with previous studies. The severity of insomnia worsens with increasing anxiety symptoms in populations with poorer emotional regulation (45). Thus, schizophrenics patients are at higher risk of experiencing anxiety and depression compared to the general population (46). The reason for this is that, firstly, anxiety can lead to excessive rumination and worry, making it difficult to fall asleep at night or causing frequent awakenings (47). Secondly, symptoms of depression, such as feelings of low mood and loss of interest, can disrupt normal sleep patterns and structures, leading to early waking or excessive sleep. Furthermore, anxiety and depression may increase patients’ sensitivity to negative stimuli, further exacerbating sleep disturbances (48). Overall, the presence of these symptoms creates a vicious cycle that worsens the sleep problems of schizophrenia patients, thus affecting their daytime functioning and quality of life (15, 49, 50). Therefore, healthcare workers should pay attention to the negative emotional experiences of schizophrenia patients, such as anxiety and depression, and provide follow-up visits and emotional counseling to patients with these characteristics. Similarly, we learn that there is a significant negative relationship between the level of social support obtained by schizophrenic patients and anxiety depression via the study results, which demonstrates the importance of a good level of social support in maintaining a well-being psychological state among schizophrenia patients, as reported in other studies. In addition, based on the negative relationship between social support and anxiety and depression, and the positive relationship between anxiety and depression and insomnia, we can reasonably propose our research goal: the important role of anxiety and depression in the effect of social support on sleep.

Social support has been shown to indirectly influence sleep quality through the regulation of mental health in populations such as adolescents, healthcare workers, and stroke patients (29, 31, 33). Similarly, our study reveals the same pattern in schizophrenia patients, validated by hierarchical multiple regression analyses and serial mediation model. The study demonstrated that anxiety and depression play a fully mediating role between social support and sleep quality, with anxiety and depression having a significant mediating effect on sleep quality through three specific pathways. The pathway “social support → depression → sleep quality” had the highest proportion of effects. Depression is strongly associated with decreased sleep quality, which is proven by extensive research (51, 52). High levels of social support is recognized as an important external resource for promoting physical and mental health, providing emotional comfort, practical help, and cognitive support, thus reducing patients’ depressive symptoms (53). With the reduction of depressive symptoms, patients’ negative emotions and thoughts decreased, and sleep patterns and structures gradually returned to normal, which in turn improved sleep quality. Thus, social support had the most significant effect on sleep quality through the mediating variable of depression. The findings also raise important implications for clinical practice. Specifically, interventions that only target sleep disorders in specific groups, such as schizophrenia, may not produce optimal outcomes if they ignore broader psychological and social factors. As such, a more comprehensive approach is warranted to evaluate and address the psychosocial well-being and sleep quality of patients with schizophrenia.

## 5 Conclusion and suggestions

In the context of the high prevalence of poor sleep in schizophrenia patients, this study explored the relationship between social support, anxiety, depression, and sleep in schizophrenia patients, confirming the fully mediating role of anxiety and depression in the relationship between social support and sleep. These findings highlight the complex mechanisms by which social support affects sleep quality by addressing underlying mental health problems, and also further refine previous research findings on the detrimental effects of anxiety and depression on sleep quality in different groups. Social support can indirectly affect sleep quality in schizophrenia patients via anxiety and depression. Specifically, increasing the level of social support has the potential to reduce symptoms of anxiety and depression, improving sleep quality in schizophrenia patients. This study may provide potential insights and guidance for the development and implementation of intervention strategies and measures to improve sleep quality in schizophrenia patients.

Our findings advocate the development and implementation of diverse interventions aimed at increasing levels of social support, reducing symptoms of anxiety and depression, and, through these means, improving sleep quality. Further research should endeavor to explore the specific effects of different types and sources of social support on the quality of sleep in people with schizophrenia and how these supports can be effectively integrated into existing treatment and care practices. In addition, the results of this study emphasize the importance of interdisciplinary collaboration, given the significant impact of psychosocial factors on sleep quality in schizophrenia patients. Coordinated efforts among mental health professionals, social workers, community-based organizations, and family members are essential for the development and implementation of effective support plans. Through this collaboration, a comprehensive supportive environment can be created to help individuals with schizophrenia improve their sleep quality and their overall quality of life. Finally, this study highlights the necessity of public health education. By raising awareness of schizophrenia and its associated sleep problems among the public, healthcare providers, and policymakers, more effective resource allocation and support strategies can be developed to meet the complex requirements of people with schizophrenia.

## 6 Limitations

Our study also has some limitations. Firstly, the subjects of this study were only from the community of Chengdu, which may limit the generalizability of our findings. Secondly, the variables such as “social support,” “anxiety,” “depression,” and “sleep” were all subjectively reported by the participants, which could lead to information bias. The hierarchical multiple regression and Baron and Kenny’s four-step mediation effects test methods assume that the relationships between variables are linear. If in reality there are nonlinear relationships between variables, this method may not accurately identify and interpret these relationships, leading to inaccurate or confusing results. In addition, multiple tests in the four-step approach may also increase the risk of type I errors (false positives). Fourth, the relationship between social support and sleep quality is complex. In addition to the symptoms of anxiety and depression, there may be other mediating variables. Future research should focus on identifying these mediating variables to more systematically and clearly understand the pathways through which social support affects sleep quality.

## Data availability statement

The raw data supporting the conclusions of this article will be made available by the authors, without undue reservation.

## Ethics statement

The studies involving humans were approved by the Ethics Committee of West China Fourth Hospital, Sichuan University. The studies were conducted in accordance with the local legislation and institutional requirements. Written informed consent for participation in this study was provided by the participants’ legal guardians/next of kin.

## Author contributions

XinL: Writing – original draft, Writing – review & editing, Conceptualization, Data curation, Formal analysis, Investigation, Validation. CL: Funding acquisition, Resources, Supervision, Writing – review & editing, Project administration. XC: Project administration, Writing – review & editing, Data curation, Investigation. FT: Data curation, Writing – review & editing, Investigation. JL: Data curation, Writing – review & editing, Software, Validation. YL: Data curation, Writing – review & editing, Funding acquisition, Methodology, Project administration, Resources. XiaL: Data curation, Writing – review & editing, Funding acquisition, Methodology, Resources. XY: Data curation, Investigation, Writing – review & editing, Formal analysis. XW: Data curation, Writing – review & editing. CZ: Data curation, Writing – review & editing, Investigation. CH: Writing – review & editing, Funding acquisition, Methodology, Resources, Supervision.
