# Supplementary material for: Social support and sleep quality in people with schizophrenia living in the community: the mediating roles of anxiety and depression symptoms
Source: Front Public Health. 2024 Jul 30;12:1414868. doi: 10.3389/fpubh.2024.1414868 (PMC11319290; doi:10.3389/fpubh.2024.1414868)
Supplement: Supplementary file 1 [file Presentation_1.pdf]

# Social support and sleep quality in community schizophrenics: The mediating roles of anxiety and depression symptoms

## Supplementary Material

Xin Liu[1], Chao Li[2], Xushu Chen[1], Fengxiang Tian[1], Juan Liu[4], Yuanyuan Liu[3], Xiang Liu[3], Xiaolan Yin[3], Xiangrui Wu[3], Chuanlong Zuo[3] , Changjiu He[2*]

[1]School of Nursing, Chengdu Medical College, Chengdu, China
[2]The Clinical Hospital of Chengdu Brain Science Institute, MOE Key Laboratory for Neuroinformation, University of Electronic Science and Technology of China, Chengdu, China
[3]Department of Epidemiology and Biostatistics, West China School of Public Health and West China Fourth Hospital, Sichuan University, Chengdu, Sichuan, China
[4]Western Theater General Hospital, Chengdu, China

*** Correspondence:**
Corresponding Changjiu He[2*]
51902943@qq.com

In order to further test the robustness of the mediating effect, we randomly selected 50% and 70% of the samples from the dataset as a new set sum to test the mediating effect, and the results are shown in Table S1 and Table S2, which are consistent with our previous findings, confirming that anxiety and depression act as a complete chain mediator between social support and sleep.

Table S1 Mediation analysis of social support and sleep quality (N = 569)

| Path | | Effect | Boot SE | 95%CI LL | 95%CI UL | Effect ratio |
|---|---|---|---|---|---|---|
| Total effect | | -0.092 | 0.029 | -0.149 | -0.035 | |
| Direct effect | | 0.024 | 0.023 | -0.021 | 0.070 | |
| Indirect effect | Total indirect effect | -0.116 | 0.018 | -0.151 | -0.083 | 100% |
| | Social support→ anxiety → sleep quality | -0.017 | 0.008 | -0.034 | -0.004 | 14.7% |
| | Social support→depression → sleep quality | -0.062 | 0.013 | -0.088 | -0.038 | 53.0% |
| | Social support→ anxiety →depression→sleep quality | -0.038 | 0.010 | -0.058 | -0.019 | 32.3% |

SE, Standard Error; CI, Confidence interval; LLCI, Lower Limit Confidence interval; ULCI, Upper Limit Confidence interval;

Table S2 Mediation analysis of social support and sleep quality (N = 788)

| Path | | Effect | Boot SE | 95%CI | | Effect ratio |
|---|---|---|---|---|---|---|
| | | | | LL | UL | |
| Total effect | | -0.088 | 0.023 | 0.000 | -0.133 | |
| Direct effect | | 0.012 | 0.018 | -0.024 | 0.049 | |
| Indirect effect | Total indirect effect | -0.100 | 0.013 | -0.127 | -0.075 | 100% |
| | Social support→ anxiety → sleep quality | -0.020 | 0.007 | -0.034 | -0.009 | 19.8% |
| | Social support→depression → sleep quality | -0.050 | 0.010 | -0.069 | -0.031 | 49.5% |
| | Social support→ anxiety →depression→sleep quality | -0.031 | 0.007 | -0.045 | -0.018 | 30.7% |

SE, Standard Error; CI, Confidence interval; LLCI, Lower Limit Confidence interval; ULCI, Upper Limit Confidence interval;
